# Transmission of beneficial yeasts accompanies offspring production in *Drosophila*—An initial evolutionary stage of insect maternal care through manipulation of microbial load?

**DOI:** 10.1002/ece3.10184

**Published:** 2023-06-17

**Authors:** Hanna Cho, Marko Rohlfs

**Affiliations:** ^1^ Institute of Ecology, Insect and Chemical Ecology Group University of Bremen Bremen Germany

**Keywords:** crop, *Drosophila melanogaster*, *Hanseniaspora uvarum*, microbe transmission, mutualism, parental care, yeast

## Abstract

Parent‐to‐offspring transmission of beneficial microorganisms is intimately interwoven with the evolution of social behaviors. Ancestral stages of complex sociality–microbe vectoring interrelationships may be characterized by high costs of intensive parental care and hence only a weak link between the transmission of microbial symbionts and offspring production. We investigate the relationship between yeast symbiont transmission and egg‐laying, as well as some general factors thought to drive the “farming” of microscopic fungi by the fruit fly *Drosophila melanogaster*, an insect with no obvious parental care but which is highly dependent on dietary microbes during offspring development. The process of transmitting microbes involves flies ingesting microbes from their previous environment, storing and vectoring them, and finally depositing them to a new environment. This study revealed that fecal materials of adult flies play a significant role in this process, as they contain viable yeast cells that support larval development. During single patch visits, egg‐laying female flies transmitted more yeast cells than non‐egg‐laying females, suggesting that dietary symbiont transmission is not random, but linked to offspring production. The crop, an extension of the foregut, was identified as an organ capable of storing viable yeast cells during travel between egg‐laying sites. However, the amount of yeast in the crop reduced rapidly during periods of starvation. Although females starved for 24 h deposited a smaller amount of yeast than those starved for 6 h, the yeast inoculum produced still promoted the development of larval offspring. The results of these experiments suggest that female *Drosophila* fruit flies have the ability to store and regulate the transfer of microorganisms beneficial to their offspring via the shedding of fecal material. We argue that our observation may represent an initial evolutionary stage of maternal care through the manipulation of microbial load, from which more specialized feedbacks of sociality and microbe management may evolve.

## Graphical abstract

Parent‐to‐offspring transmission of beneficial microorganisms is intimately interwoven with the evolution of social behaviors. We investigate the relationship between yeast symbiont transmission and egg laying, as well as some general factors thought to manipulate the microbe management of the fruit fly *Drosophila melanogaster*, an insect with no obvious parental care but which is highly dependent on dietary microbes during offspring development. The results from this research suggest that female *Drosophila* fruit flies have the ability to store and regulate the transfer of microorganisms beneficial to their offspring via the shedding of fecal material.

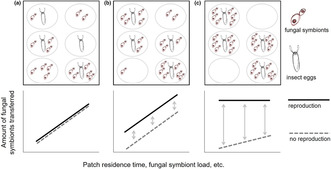

## 1 INTRODUCTION

Animal life cycles are closely linked to interactions with microorganisms, which include lethal pathogens but also essential mutualists (McFall‐Ngai et al., 2013). Control over the microbial environment is therefore crucial for animal fitness (Douglas, 2020). Consequently, natural selection has favored a wealth of “management tools” at the molecular, cellular, and behavioral levels, which allow animals to promote the growth of beneficial ones and keep pathogenic microbes in check (Cremer & Sixt, 2009; Kappeler et al., 2015; McFall‐Ngai, 2007). The selective and directed transfer of beneficial microbes from parents to offspring is necessary for many insect taxa to colonize and exploit otherwise inaccessible environments (Feldhaar, 2011; Klepzig et al., 2009). The effort associated with the transmission of extracellular symbionts across generations is also commonly referred to as a form of parental care (Archie & Tung, 2015; Troyer, 1984; Trumbo, 2012). Parental care is thought to be the precursor of complex, transgenerational social behaviors expressed in small family groups (Wilson, 1975). Intensive parental care that refines microbe transmission and in turn promotes offspring performance can start a positive feedback loop between microbe management and sociality (Biedermann & Rohlfs, 2017). In other words, the expression of costly social traits associated with microbe detection, maintenance, and vectoring are outweighed by fitness benefits resulting from maintaining symbiotic relationships.

Sophisticated microbe management in fungus‐farming insects is often closely interwoven with sociality, which is particularly evident in obligate eusocial ants and termites and several lineages of facultative eusocial wood‐boring weevils (Biedermann & Taborsky, 2011; Hölldobler & Wilson, 2011; Jordal et al., 2011). Comparable to human agriculture, fungus‐farming in insects involves (i) the transmission and propagation of the fungal symbiont, aka “sowing,” (ii) processes that promote the growth of the symbiont, aka “cultivation,” and (iii) the consumption of the cultivated fungus, aka “harvesting” (Mueller et al., 2005). A stable environment and continuous resource availability favor partner fidelity in symbiotic interactions, as well as investment in social behavior (Batstone et al., 2018; Korb & Heinze, 2016). It is therefore expected that strong links between the cultivation of specific fungi and social behavior are found where insects colonize comparatively long‐lived environments such as wood (e.g., ambrosia beetles; Diehl et al., 2022) or where the insects themselves create stable environmental conditions (e.g., ants and termites; Biedermann & Vega, 2020). In highly fluctuating environments, where the survival chances of offspring depend heavily on external factors that cannot be controlled by adult insects, the costs of social behavior may exceed their benefits. One would therefore not expect to find particularly pronounced social behavior here but rather observe traits that correspond to preliminary stages of a positive feedback loop between microbe management and sociality, for example, quite simple insect‐fungus mutualisms that are fostered by primal parental care (Biedermann & Rohlfs, 2017). Here, we expand the idea of the mutualism‐sociality feedback by formulating hypothetical outcomes of the interconnections between the transfer of symbiont fungi, offspring production, and other factors that likely influence the shedding of viable fungal material from adult insect hosts. We propose a continuum in which the correlation between fungal transmission and offspring production becomes progressively stronger, independent of factors such as the duration of residence in a patch or the microbial load of transmitting insects.

**FIGURE 1:**
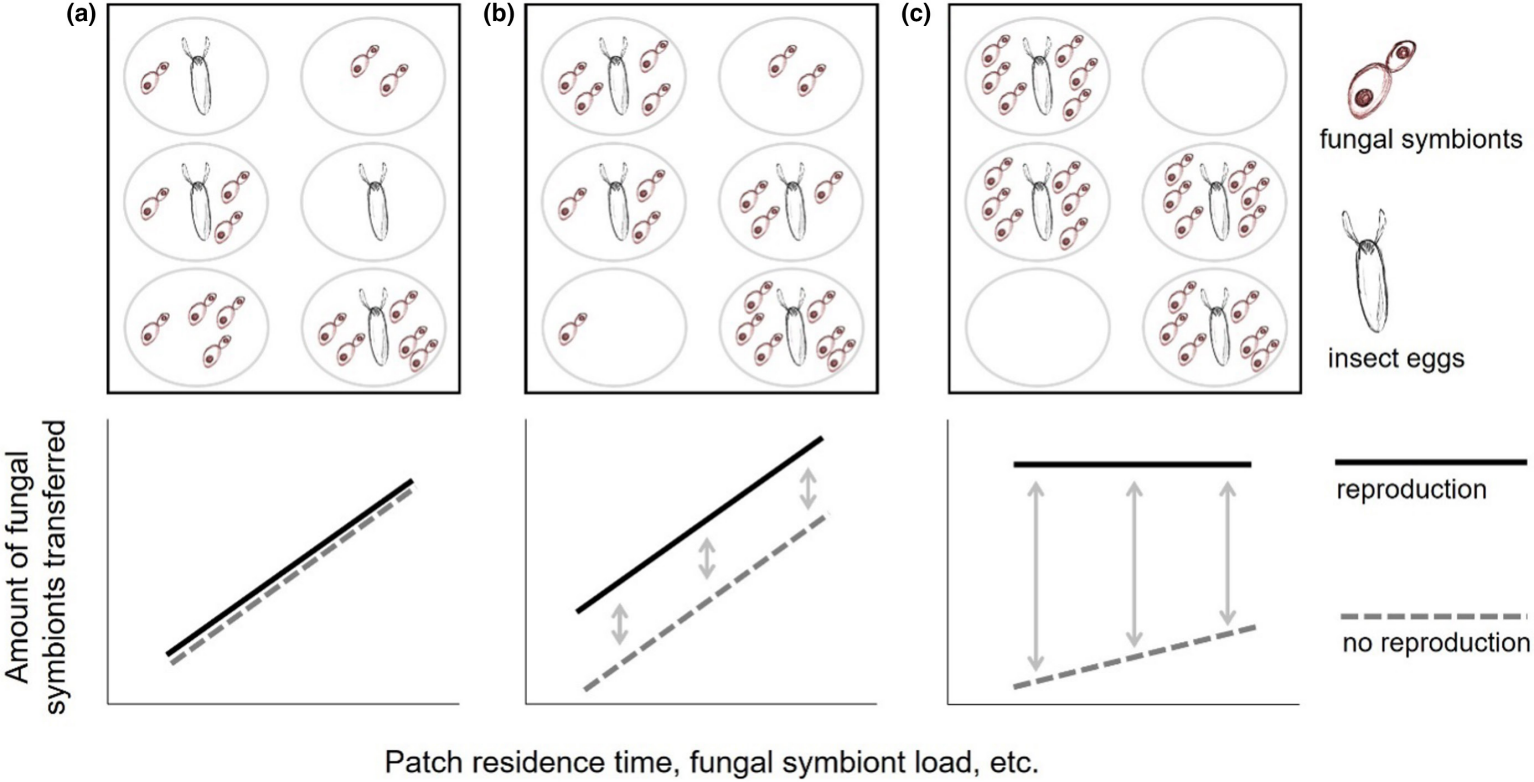
Parent‐to‐offspring fungal symbiont transmission continuum, ranging from no to advanced control of the fungal microbiota by insects. (a) Female insects have no control over the transmission of fungal tissue. Transmission depends primarily on residence time at a breeding site, with fungal cells shedding from the body surface at random. There is no reproduction‐dependent control over the pathways that mediate the transmission of fungal material. Therefore, the presence of symbionts is not correlated with the presence of insect progeny. (b) Some behaviors related to offspring production enhance symbiont transmission, for example, targeted body surface rubbing, and increased or altered defecation. Nevertheless, high variability still dominates symbionts transfer, for example, the amount of fungal material carried by adults and their residence time. Besides moderately developed modes of transfer, random effects still dominate the transfer of fungal material, but some behaviors involved in the transfer of fungal material are better controlled. Weak correlations between symbionts and insect progeny can be expected (c) Transmission of fungal symbionts offspring‐producing females is independent of residence time and associated nonreproductive activities. A fungal inoculum is transferred specifically for the purpose of supplying the offspring with symbionts. The prerequisite is mechanisms of targeted storage and transfer, for example, controlled emptying of a storage organ, which is synchronized with the production of offspring. The supply of offspring with symbionts is strongly coupled with selected traits that mediate effective symbiont transfer. There is a strong spatial correlation between insect progeny and the presence of symbionts.

The hypothetical continuum ranges from random microbial “footprints” containing viable fungal material, which insects leave during patch visits (null hypothesis) to a more directed transfer of a dedicated set of fungal symbionts (Figure 1). At the start of the continuum, we expect to see no control over the transfer of fungal symbionts resulting in a complete random transmission strongly affected by patch residence time associated with random shedding from the body surface (Figure 1a). Then, the increase in transmission due to developed behaviors such as dedicated rubbing of the body surface or enhanced defecation decouples the factors associated with reproduction from the factors associated with random shedding. High variability is observed due to random shedding still dominating over any other factors (Figure 1b). As the transmission of fungal tissue becomes higher in offspring‐producing females, the transfer becomes increasingly independent of residence time, and other factors such as fungal symbiont load, the latter may be better controlled by more efficient storage of fungal symbionts and more dedicated paths of transmission (Figure 1c).

To investigate these theoretical considerations, we explore the *Drosophila*‐yeast mutualism as a model of the evolution of mutualism‐sociality feedbacks. Yeast fungi have an important function in the *Drosophila* life cycle. They provide essential nutrients (Yamada et al., 2015) and stabilize habitat conditions in the face of parasites and pathogens in ephemeral fruit substrates (Anagnostou et al., 2010; Caballero Ortiz et al., 2018). Volatile chemicals released by yeasts contribute significantly to establishing contact with both adult and larval fruit flies (Becher et al., 2012; Stötefeld et al., 2015) and may thus guide flies to high‐quality breeding and feeding sites. Again, flies are known to transmit yeast cells that can permanently alter the microbiota of the developmental site of their offspring (Buser et al., 2014; Stamps et al., 2012). However, because of no unambiguous evidence that yeasts have coevolved with fruit flies or other insects, the origin of the putative mutualism between *Drosophila* and yeasts is a subject of current debate (Günther et al., 2019; Günther & Goddard, 2019; Hoang et al., 2016). Flies may only associate randomly with yeasts, using volatile cues that are “unintentionally” emitted by the fungi (Günther & Goddard, 2019) therefore, the shedding of fungal propagule by egg‐laying flies may not be due to specialized mechanisms but also just a coincidence (our null hypothesis, Figure 1). To test this hypothesis, we used an individual‐based experimental approach that correlates yeast transmission with inter‐individual variation in offspring production, body size, breeding site (patch) residence time, and yeast load of female *D. melanogaster* flies. Moreover, the ability to transmit symbionts requires mechanisms that allow the storage and transport of viable microbiota (Douglas, 2020; Hosokawa & Fukatsu, 2020). In *Drosophila*, coprophagy appears to be the main route of transmission of external symbionts to the offspring, that is, the flies defecate on the breeding site and the larvae take up the symbionts and spread them further in the breeding substrates (Ma & Leulier, 2018; Pais et al., 2018). Besides the gut itself, a sack‐like, expandable extension of the flies' foregut, the crop (Miguel‐Aliaga et al., 2018), may function as a potential storage organ for symbiont fungi (Pais et al., 2018; Stoffolano & Haselton, 2013). Despite the potential of the crop of the fruit fly as a storage organ for symbiont fungi, the extent to which reproductive status and variation in hunger may influence its capacity to hold yeast and the subsequent impact on the microbial load transmitted is poorly understood. Therefore, we investigated the capacity of the crop of female *D. melanogaster* to serve as a reservoir for symbiotic yeast fungi that are passed to their offspring via fecal matter deposition. With this study, we aim to shed light on possible scenarios of the onset of insect‐microbe mutualism driven by maternal transmission of microbes to offspring.

## 2 MATERIALS AND METHODS

### 2.1 Insect cultivation

The *Drosophila melanogaster* population used originated from 113 female flies collected in Kiel, Germany (~54°N, 10°E) in August 2006 (Wölfle et al., 2009). The population has been kept at a size of more than 200 flies per generation to maintain the outbreed properties. The insects were kept in a ventilated plastic cage (39 × 28 × 28 cm) with access to water and an artificial rearing medium at 20 ± 1°C (Wölfle et al., 2009). The artificial medium for rearing was prepared by combining two mixtures. Mixture 1 was prepared with 12 g agar and 50 g sugar in 500 mL distilled water, which was heated up in a microwave until boiling. After cooling down, 10 mL sorbic acid (10% [w/v] in Ethanol) and 10 mL nipagin (10% [w/v] in Ethanol) were added and stirred thoroughly. Mixture 2 was prepared with 250 g applesauce, 50 g corn flour, and 70 g brewer's yeast in 100 mL distilled water. Both mixtures 1 and 2 were mixed well and were poured into a vial for use in rearing. To create a semi‐natural breeding environment in which symbiotic microbiota become essential for the flies' reproductive success prior to the experiment a subpopulation of our fly culture was kept on raspberries instead of an artificial medium for three generations. We used frozen raspberries purchased from a local supermarket. We did not add any specific microorganisms but let the flies get associated with those already present on the fruits and their standard culture environment.

#### 2.1.1 Yeast transmission by individual *Drosophila melanogaster* females

**FIGURE 2:**
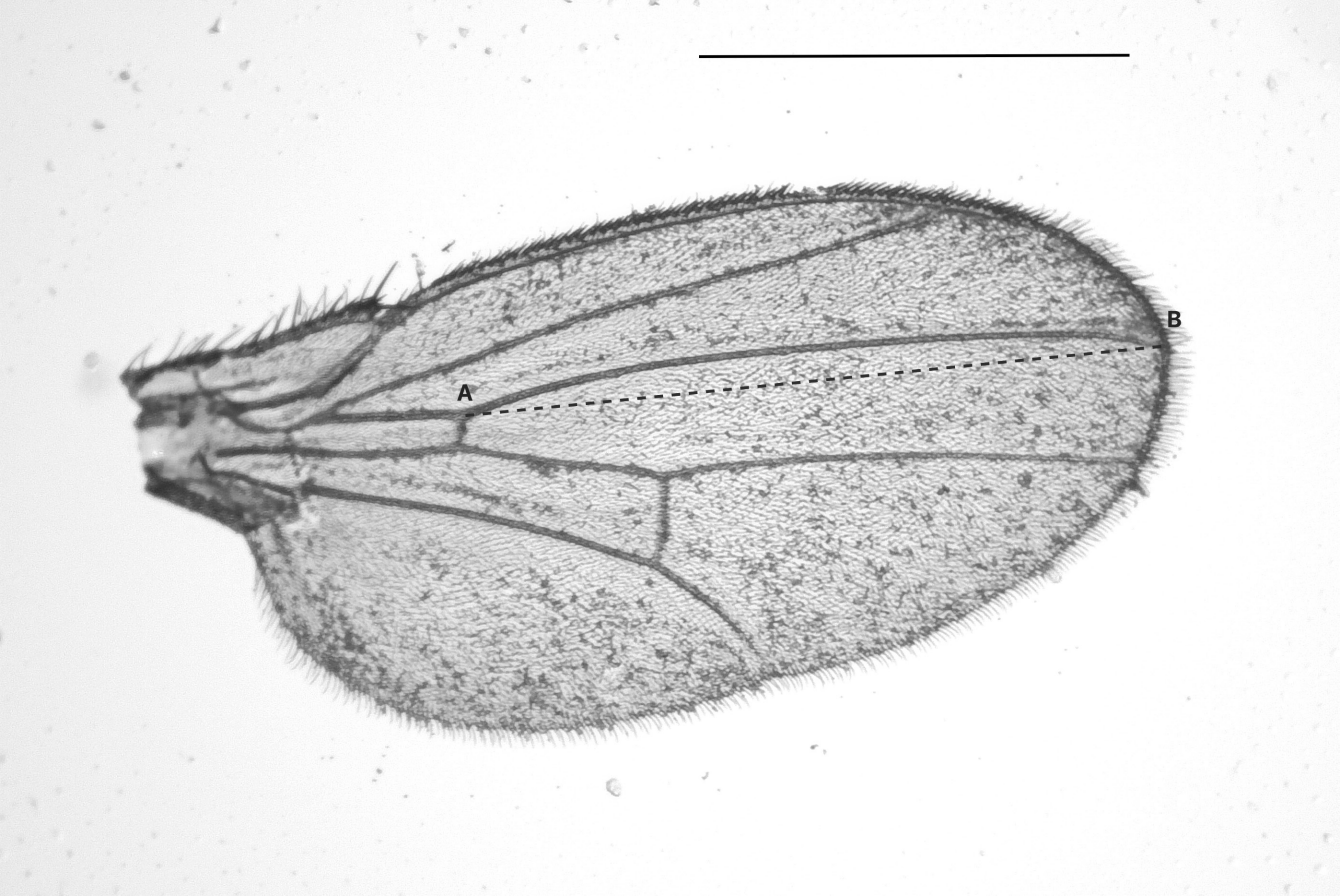
*Drosophila melanogaster*; right wing, dorsal view, line A–B was measured (software Zen2) as a proxy to body size. Scale bar = 1 mm.

A semi‐natural breeding substrate was created to test the influence of body size, microbial load, patch residence time, and egg‐laying of individual females on the transmission of yeast fungi. To this end, a raspberry medium was prepared by extracting juice from store‐bought frozen raspberries. The fruit was blended and sieved three times before being microwaved for sterilization, and subsequently mixed with distilled water for a final ratio of 1:1 (juice to water). Agar (3 g per 1 L) was added to the juice‐water mixture and was heated to solidify the medium. 750 μL of the raspberry medium were filled in 20 mL plastic cups (3 cm diameter, 2 cm height), which were placed inside a plastic vial (3.5 cm diameter, 8 cm height); the vials were sealed with foam stoppers. Individual *D. melanogaster* females between the age of 5–10 days from the previously described population cages were introduced to these experimental units. For a duration of 6 days, 91 mature female flies were observed for their residence time on their first patch visit, during the evening hours from 5 p.m. to 10 p.m. on each day of the observation. Control patches without flies were also left for observation to assess the level of random settlement of yeasts. Ten vials were observed at a time and were replaced with a new sample once the fly completed the visit. The patch residence time was calculated from the time when the flies entered and left the patch. To avoid recording residence times that are unlikely to be due to flies being arrested there by properties of the patch, the residence time was only established if the patch visit lasted for at least 30 s. As soon as the flies left the patch they were removed from the vials and put into fresh tubes inside an ice basin. A wing was removed from paralyzed individuals under the stereomicroscope and the length was measured and used as a proxy for their body size (Figure 2; Wolf et al., 2000). Then, flies were individually crushed in 1 mL saline (NaCl: 0.8%, Tween‐80: 0.01%) with a pestle in 2 mL tubes to collect yeast cells in/on the body as a proxy to yeast load of a fly (yeast load). The number of eggs laid by individuals was counted before each patch was soaked and shaken with saline. The raspberry medium disintegrated in saline and the resulting suspension was collected per patch (patch‐yeast). The track dilution technique was used (Jett et al., 1997) for the quantification of the yeast transmission to the patch and the amount of yeast flies still carried. For this, a selective yeast medium, 3 g yeast extract, 3 g malt, 5 g soy peptone, 10 g glucose, and 15 g agar in 1 L distilled water was prepared and autoclaved in advance for plating. Chloramphenicol (0.1 g/L) was also added to the solution to prevent any bacterial growth. Each suspension was pipetted onto three tracks of the plate as technical triplicates. Yeast colonies that grew from these triplicates were then combined to calculate an average. Due to variation in its viscosity, 10 μL yeast‐load suspension was pipetted per track and 25 μL patch‐yeast suspension was pipetted per track. Plates were incubated at 25°C for 2 days. Subsequently, yeast colony forming units (CFU) per mL were calculated. All steps were conducted under a laminar flow hood and all components were autoclaved to avoid contamination.

#### 2.1.2 Transmitted yeast fungi as dietary symbionts

Based on the identified yeast fungi (Appendix II, Table A9) that were transmitted by individual *D. melanogaster* females in Experiment 1, we tested whether they indeed function as supplementary dietary symbionts in the life cycle of the flies. For this, we used axenic larvae that were prepared from freshly laid eggs of *D. melanogaster* in a vial with an artificial rearing medium. The collected eggs were submerged in 30 mL of 6% sodium hypochlorite solution. A fine brush was used to dislodge the eggs from the medium before transferring the egg suspension to a 50 mL falcon tube. After 5 min, the bleached eggs were filtered through a fine filter mesh, and for the eggs that were still attached to the walls of the falcon tube 70% ethanol was used to release them into the filter. The filtered eggs were washed with 2 L distilled water before being transferred to a chloramphenicol‐glucose agar plate and incubated at 25°C overnight. The following day, the freshly hatched larvae were transferred individually into 2 mL polypropylene micro‐vials filled with 1 mL sterile raspberry medium inoculated with 10 μL of respective yeast solutions (see below) containing 500 fungal cells. The yeasts used for inoculation were cultivated in the liquid yeast medium for 2 days at 25°C in a shaker and consecutively the cells were counted using a hemocytometer to adjust the titer of the cell suspension. The following yeast isolates were used: three isolates (Appendix II, Table A9) from the experiment described above, which we tentatively identified as *Hanseniaspora uvarum* (isolate P38), *Pichia terricola* (isolate F13‐1), and *Wickerhamomyces pijperi* (isolate P51); two isolates that were previously found to be transmitted by drosophilids, *Candida californica* (isolate PYCC6277), *Starmerella bacilaris* (isolate PYCC 6281), and *Saccharomyces cerevisiae* (isolate DSM 70449), commonly known as brewer's yeast. The latter three yeast species are known to positively affect *D. melanogaster* development (Anagnostou et al., 2010; Meriggi et al., 2020; Stamps et al., 2012; Stötefeld et al., 2015). The vials were sealed with an autoclaved cotton plug (~1 cm dental roll) and incubated at 25°C, 70% humidity, and 12:12 light–dark cycle. Twenty‐four replicates per treatment were prepared in which the larvae were mono‐associated with one of the six yeast isolates. The survival of the larvae to adulthood was observed for each treatment.

#### 2.1.3 The crop as a potential storage organ of symbiotic yeast fungi

To test whether the crop has the potential to function as a storage organ for fungal symbionts, and if so, how efficiently it stores viable yeast cells that are then transferred to the next breeding patch, we manipulated the mating and hunger status of *D. melanogaster* females. We expected the size, that is, the filling, of the crop to (1) positively correlate with the amount of viable yeast cells it contains, (2) to be higher in mated females, and (3) negatively correlate with progressive fasting. While females empty the crop for their own nutritional purposes during fasting, mated and *ad libitum* fed females ready to lay eggs could primarily retain a larger number of dietary symbionts in the crop, which are gradually released into the gut to increase the number of yeast cells in the gut and thus in the feces deposited next to the eggs.

**FIGURE 3:**
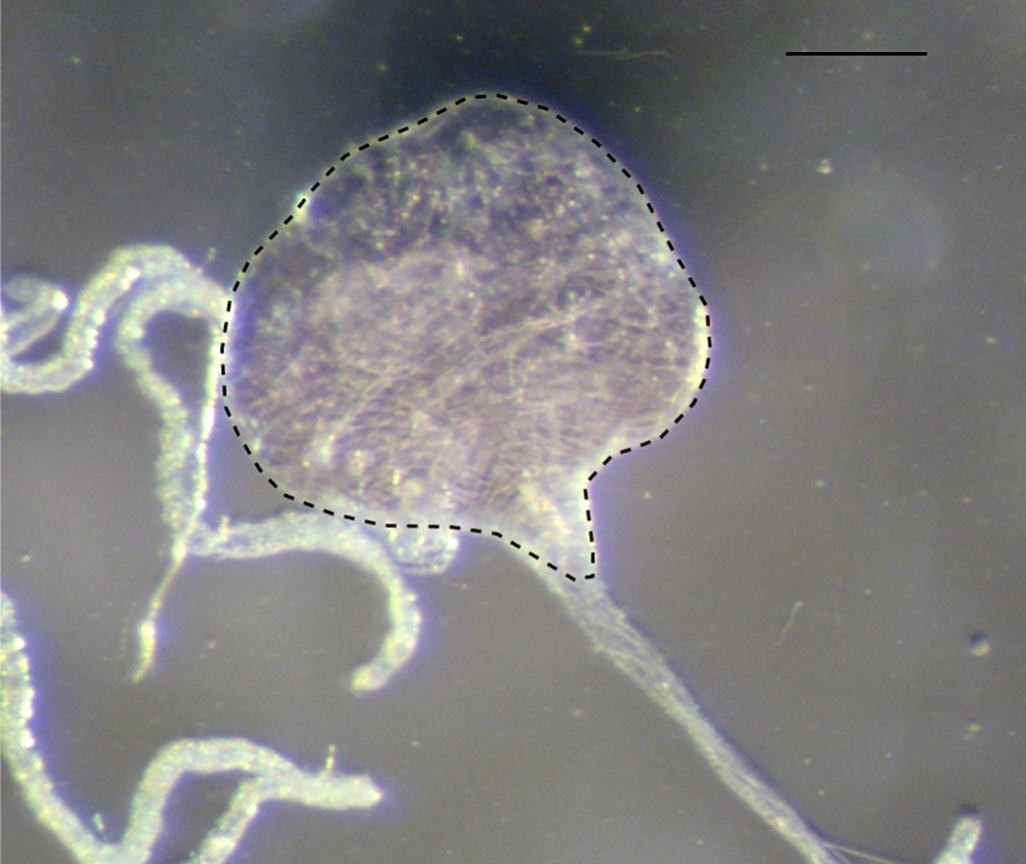
A crop pulled out of female *Drosophila melanogaster*. The selected area (dotted line) was measured in mm2 (software Zen2) to represent the size of a crop. Scale bar = 0.1 mm.

To investigate these assumptions, *D. melanogaster* flies were mono‐associated with *H. uvarum*. Based on previous studies (Chakraborty et al., 2022; Chandler et al., 2012; Hamby et al., 2012), *H. uvarum* in particular appears to be widespread and common in *Drosophila* populations, which is why we generated gnotobiotic flies with *H. uvarum* in all other experiments conducted in this study. To achieve mono–association, axenic *D. melanogaster* larvae were individually raised in 2 mL polypropylene micro–vials filled with 1 mL sterile artificial rearing medium. These vials were then sealed with cotton plugs. Upon emergence, the flies were sorted by sex and placed into separate sterilized plastic cages provided with a sterile raspberry medium that was inoculated with *H. uvarum*, along with water‐agar to facilitate the association of flies with the yeast and maturation of eggs in ovaries. Three days later, 28 females were placed in individual vials, and out of those, 14 females were introduced to males. The following day, females were transferred individually to a fresh vial with a sterile raspberry medium (see Experiment 1) for oviposition for 5 h and then removed from their vials. The number of eggs laid into the raspberry medium was counted to distinguish the egg‐laying activity between the mated and virgin females. Subsequently, all females were dissected under the stereomicroscope, mature oocytes at stage 14 (Jia et al., 2016) were counted from both ovaries, and the measurements for body size (Figure 2) and crop size (Figure 3) were taken. Each crop was then crushed in 500 μL saline and the entire suspension of 500 μL was used for the spread plating as described by Sanders (2012) on selective yeast medium (Sanders, 2012). Plates were incubated at 25°C for 2 days. The number of viable yeast colonies was counted, and CFU/mL was calculated.

To investigate how much of the yeast remains in the crop after fasting and to see whether starved females can still support the development of their offspring, 4‐day‐old females, mono‐associated with *H. uvarum*, were used. Either after 6 or 24 h of fasting, females (30 per treatment) were removed from the population cage and placed in an individual vial with a sterilized raspberry medium (see Experiment 1). Each female was allowed 2 h to oviposit and was then removed from the vial. The eggs were counted, and the surface of the patches was washed with saline solution with the egg intact for plating. Washing and plating of yeasts were done using the track dilution technique as described in Experiment 1. To also compare the developmental success of larvae that emerged from eggs laid by fasting females a second experiment with the same setting as above was conducted simultaneously, but this time the patches were not washed, and eggs were allowed to develop into the adult stage. Days to emergence and survival were recorded.

#### 2.1.4 Feces as a source of heritable symbiont fungi

**FIGURE 4:**
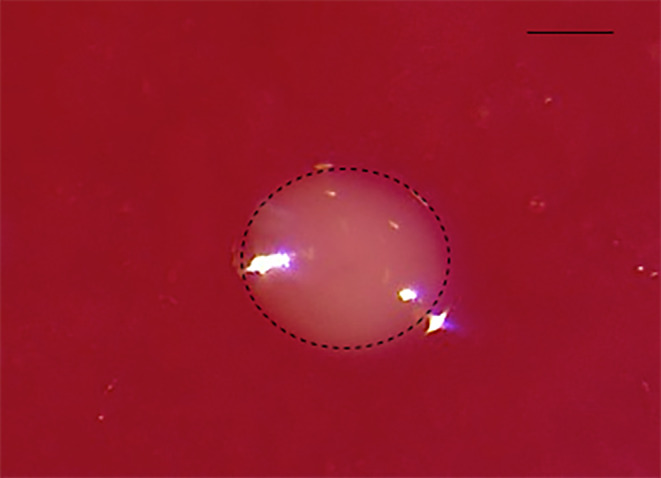
Fecal droplet on a raspberry patch excreted by the female fly. The selected area (dotted line) was measured in mm^2^ (software Zen2) to represent the size of a fecal droplet. Scale bar = 0.1 mm.

**FIGURE A1:**
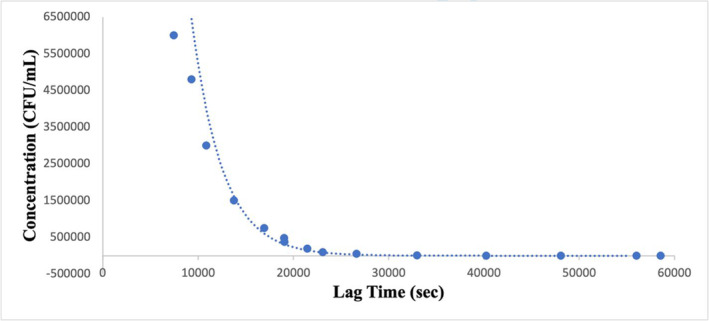
A calibration curve drawn from the different concentrations of *H. uvarum* with their corresponding lag time of population growth. Exponential equation (*y* = 116975172e‐0.000311 *x*) was then used to extrapolate the initial number of cells in each fecal droplet.

Since coprophagy is most likely the main pathway through which microbes are vertically transferred in *Drosophila*, we explicitly tested to what extent adult fecal material contains viable fungal symbionts. Out of 69 female mono‐associated flies, 35 were mated and the others remained virgins (see Experiment 3). After 4 days, flies were transferred to fresh vials with sterilized raspberry medium (see Experiment 1). The individual flies were given 5 h to interact with the substrate before they were removed. Single clearly visible fecal droplets per patch were observed under the stereomicroscope and were photographed. Images were then used to calculate the area of the fecal droplets (Figure 4). The observed droplets were then picked with a brush and diluted in 200 μL of liquid selective yeast medium described in Experiment 1. Since the track dilution technique was not sensitive enough to estimate the cell numbers in the fecal droplets, yeast cell population growth was quantified instead with a microplate reader (BioTek Epoch 2). To achieve this, 96 well plates were loaded with fecal solution samples of 200 μL/well, the optical density (OD) at 600 nm was measured over 24 h and the temperature was maintained at 25°C. The measurements at the end of the analysis included a lag time for each sample, a good indicator of the initial microbial load in a sample (Bertrand, 2019). Additionally, the initial number of yeast cells in the droplets was extrapolated from the equation formulated from the lag time of the known concentration of *H. uvarum* (Appendix III, Figure A1).

### 2.2 Statistical analyses

The amount of yeast transmitted to a patch as a function of the number of eggs oviposited, the size of a fly, and the yeast load of a fly, a generalized linear model with Gaussian family with an identity link function was used and Type II ANOVA with F test was performed. The presence or absence of oviposition was additionally considered a categorical factor in the evaluation. The response variable in the linear regression model (amount of yeast transferred) was log‐transformed to meet assumptions of normality in the residuals. Additionally, the independent variables of yeast load of a fly, residence time, and the number of eggs oviposited, were log‐transformed to improve the scale and fit of the models. For the survival of larvae to adulthood on different inoculum of yeasts, data were fitted with a generalized linear model with binomial error distribution with a logit‐link function. The yeast transmission by fasting females, yeast load in crops, data were fitted with a generalized linear model with quasi‐Poisson error distribution with a log‐link function to correct for over‐dispersed data. To analyze the proportion of larvae surviving to adulthood with the yeast transmitted by the individual female on different fasting regimes, data were fitted with a generalized linear model with quasi‐binomial error distribution with a logit‐link function to correct for under‐dispersed data. For the size of the crop and the size of the fly, data were analyzed with a generalized linear model with Gamma error distribution with a log‐link function. Minimum adequate models (MAMs) were selected for all datasets, by comparing the *p*‐values of each additive and interactive term (Type II ANOVA). Insignificant terms with *p*‐values >.05 were eliminated until all terms remaining were significant (Full models & MAMs, Appendix I, Tables A1, A2, A3, A4, A5, A6, A7, A8). Model assumptions were verified through inspection of diagnostic residual plots to ensure normality and homoscedasticity of variance. All statistical analyses were conducted using R (software version 4.1.1) and R studio (version 2021.09.0).

## 3 RESULTS

### 3.1 Yeast transmission by individual *Drosophila melanogaster* females

**FIGURE 5:**
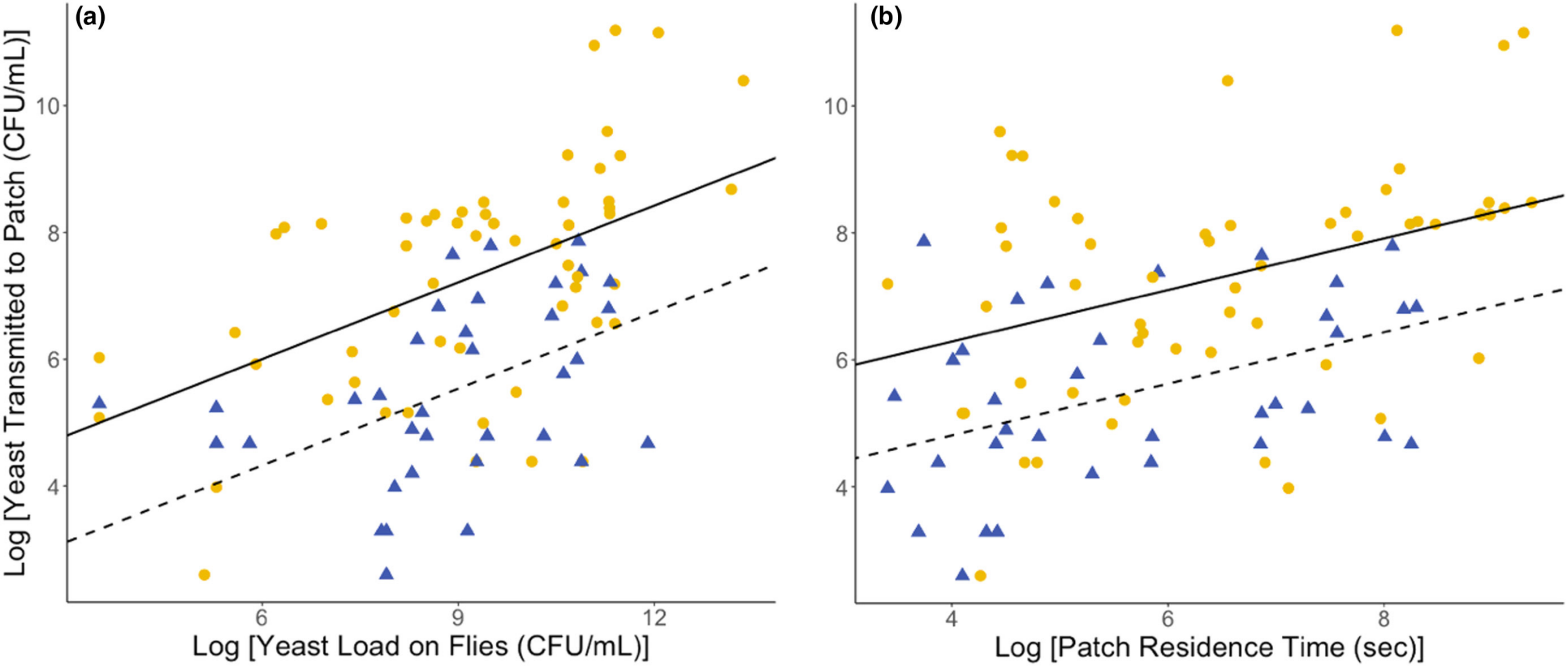
The amount of yeast transmitted by individual *Drosophila melanogaster* females as a function of the yeast load of individual flies (a) and as a function of their patch residence time (b). The yellow points represent females that oviposited, and the blue triangles represent those that did not oviposit. The solid and the dashed lines represent the results of the linear regression model for mated and virgin females, respectively.

The amount of yeast fungi transferred by individual *D. melanogaster* females during a single patch visit (Figure 5) was increased with the amount of yeast flies carry (Figure 5a; *F*_1,87_ = 38.54, *p* < .001) and the time the flies spent on the fruit patch (Figure 5b; *F*_1,87_ = 24.64, *p* < .001), that is, the yeast load of flies and their residence time. Flies that laid eggs (Figure 5a,b, solid line) deposited more yeast than those that did not lay eggs (*F*_1,87_ = 21.24, *p* < .001). However, the number of eggs laid during the patch visit and the flies' body size had no statistical significance on the amount of yeast transferred. The flies' egg‐laying activity is thus positively related to the amount of yeast transmitted when visiting a substrate patch.

### 3.2 Transmitted yeast fungi as dietary symbionts

**FIGURE 6:**
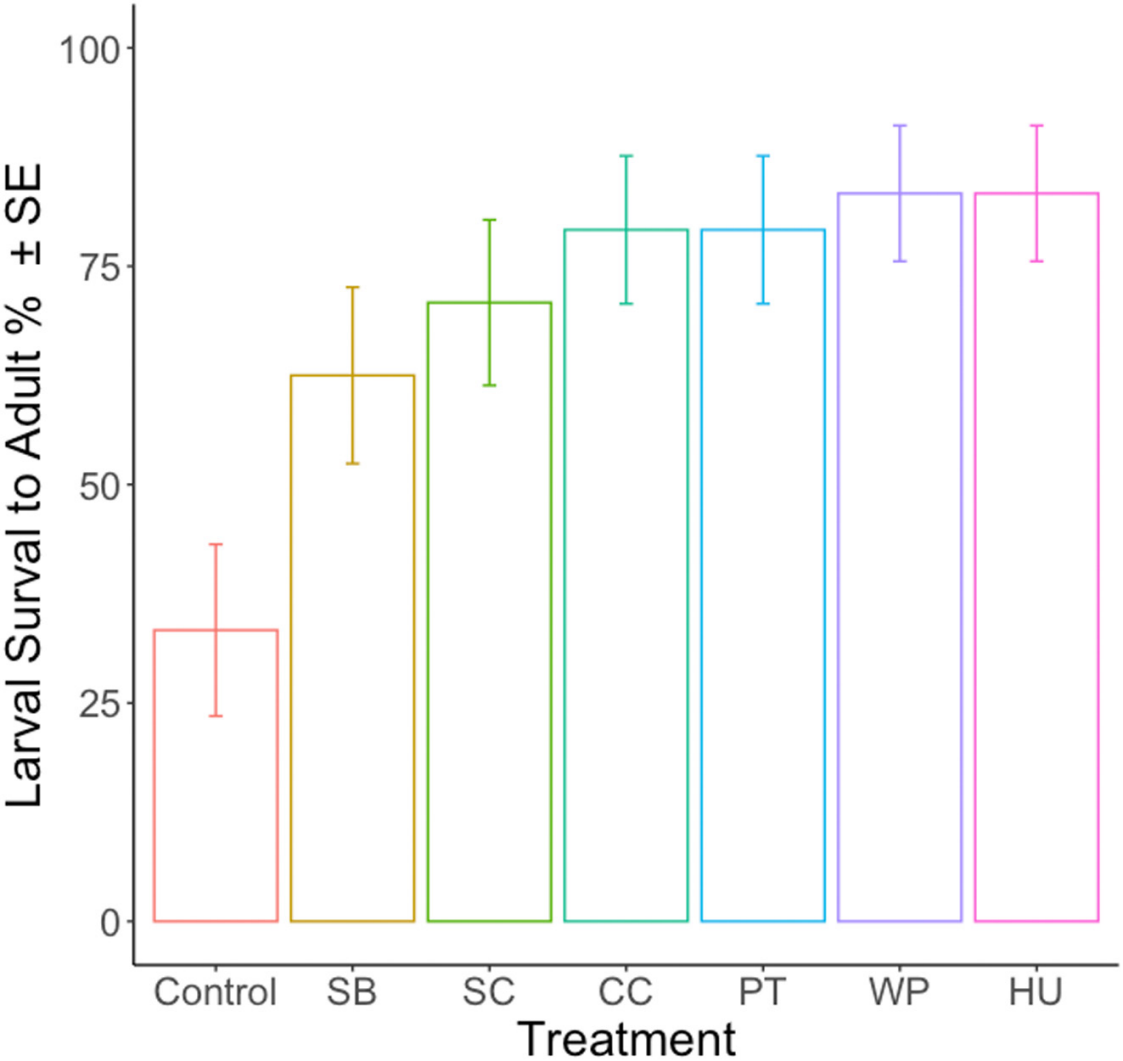
Larva to adult survival of *Drosophila melanogaster* in 6 different yeast treatments compared to the control group with no yeast inoculum. Yeasts used are as follows; *Starmerella bacilaris* (SB), *Saccharomyces cerevisiae* (SC), *Candida californica* (CC), *Pichia terricola* (PT), *Wickerhamomyces pijperi* (WP), *Hanseniaspora uvarum* (HU).

The results of the logistic regression analysis showed that there is a significant effect of yeast inoculation (Treatment) on the successful development of larvae into adult flies (Figure 6; *χ* ^2^ = 20.91, df = 6, *p* < .01). The reference level was the control group that had no yeast inoculum. These findings suggest that the different yeast species found to be transmitted by the flies all positively influenced the survival of the larvae to adulthood.

### 3.3 The crop as a potential storage organ of symbiotic yeast fungi

**FIGURE 7:**
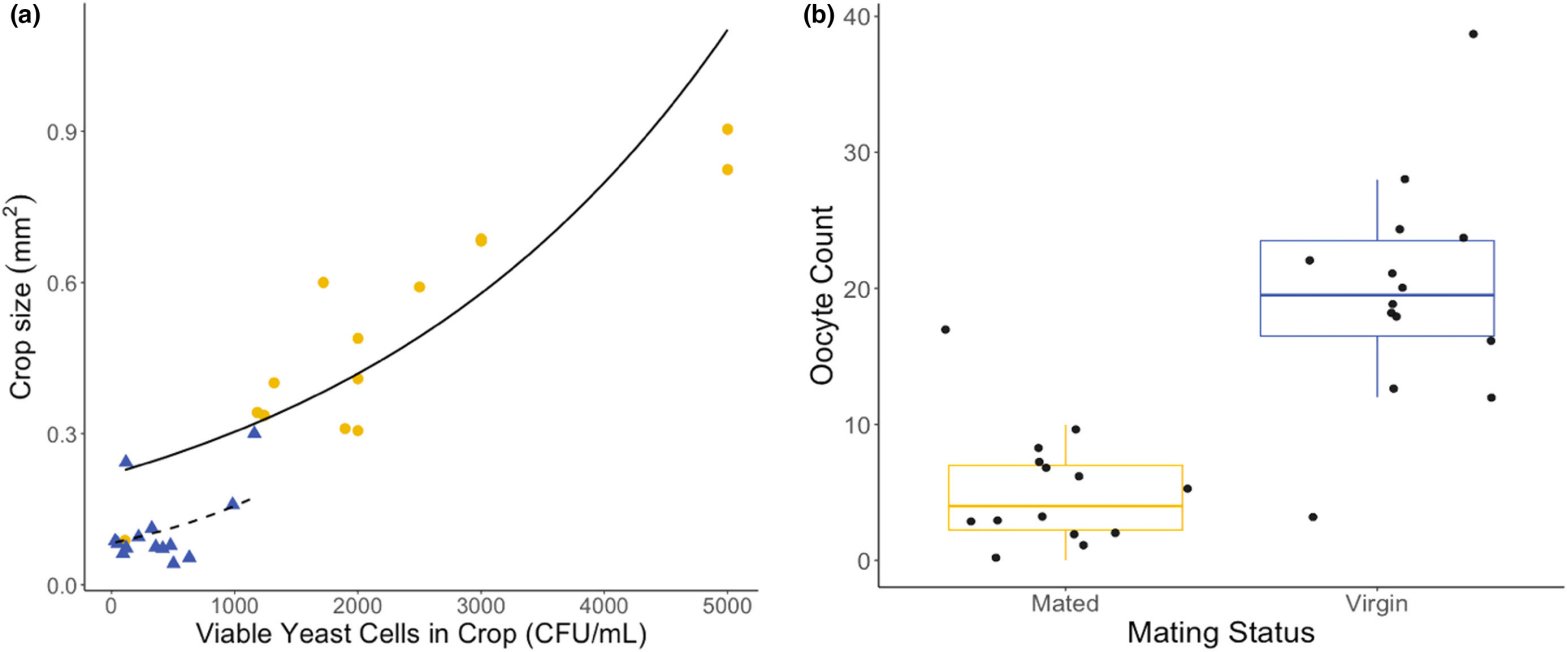
(a) Crop size as a function of viable yeast cells found in the crops of *Drosophila melanogaster* females separated by their mating status. The yellow points and the blue triangles represent the crop size of mated and virgin females, respectively. The solid and the dashed lines represent the results of the generalized linear regression model for mated and virgin females, respectively. (b) Box plot with jitter showing the oocyte counts of individual females separated by their mating status.

We found substantial variation in the size of the crops of female *D. melanogaster* (Figure 7). Although the crop is an appendage of the foregut and is located just before the proventriculus in the anterior part of the thorax, we often found the crops extended far into the abdomen of the insects. The crop size is positively related to the number of *H. uvarum* CFUs derived from the crop (*F*_1,25_ = 12.44, *p* < .01). Simultaneously, the mating status of the females significantly influenced the size of the crop (*F*_1,25_ = 8.851, *p* < .01), which ultimately influenced the amount of yeasts found in the crops. While virgin females accumulated an average of ~20 eggs in their ovaries and did not lay a single egg, mated females laid numerous eggs throughout the experiment and had an average of only ~5 eggs in their ovaries at the end of the experiment leaving possible space for the crop to expand (Figure 7b).

### 3.4 Effect of female fasting on yeast transmission and development success of the offspring

**FIGURE 8:**
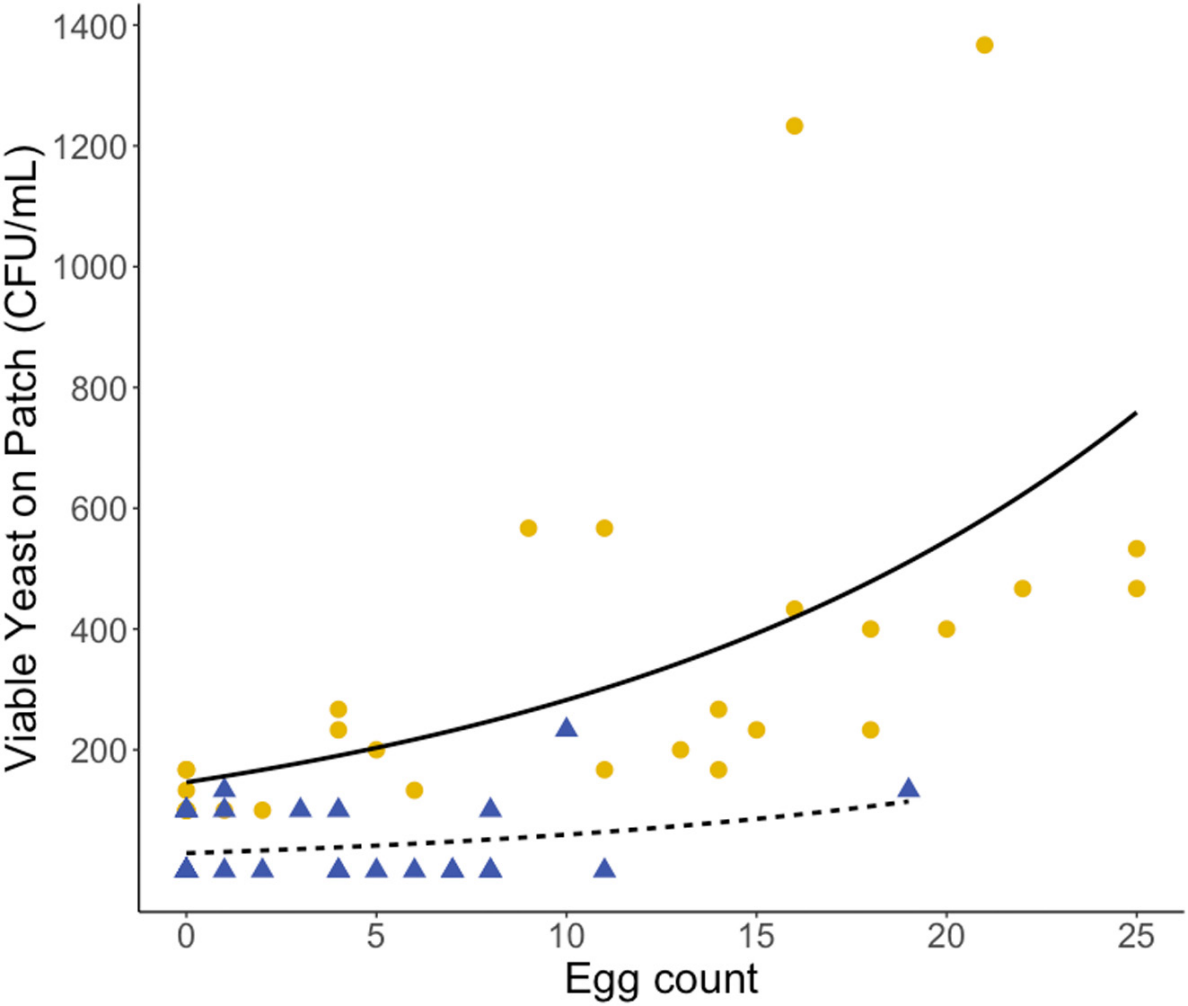
The relationship between the number of viable yeast cells transmitted to the patch to the number of eggs laid by females after the respective period of starvation. The yellow points and blue triangles represent the amount of yeast transmitted to the patch in the treatments where ovipositing females were starved for 6 and 24 h, respectively. The solid and the dashed lines represent the results of the generalized linear regression model for the 6 and 24 h starvation treatment, respectively.

Mated *D. melanogaster* females, mono‐associated with *H. uvarum* transferred significantly fewer viable yeast cells when starved for 24 h than when deprived of access to additional yeast for 6 h (Figure 8; *F*_1,57_ = 26.34, *p* < .001). Generally, the amount of yeast transferred increased significantly with the increasing number of eggs laid (*F*_1,57_ = 27.07, *p* < .001). In Experiment 1, the flies were exposed to the entire microbial community the insects could ingest from the rotting raspberries. In addition, this time we did not record the females' time spent on the patch but set a time limit of 2 h for the flies to interact with the substrate. Despite the different experimental setups, it is remarkable that under certain circumstances not only a general effect of oviposition on the shedding of dietary microbes can be observed but also a positive relationship with the quantity of the reproductive output. The number of eggs laid in the 24‐h fasting treatment was lower possibly due to females laying eggs in the water‐agar on which the flies were kept on during the period of fasting.

**FIGURE 9:**
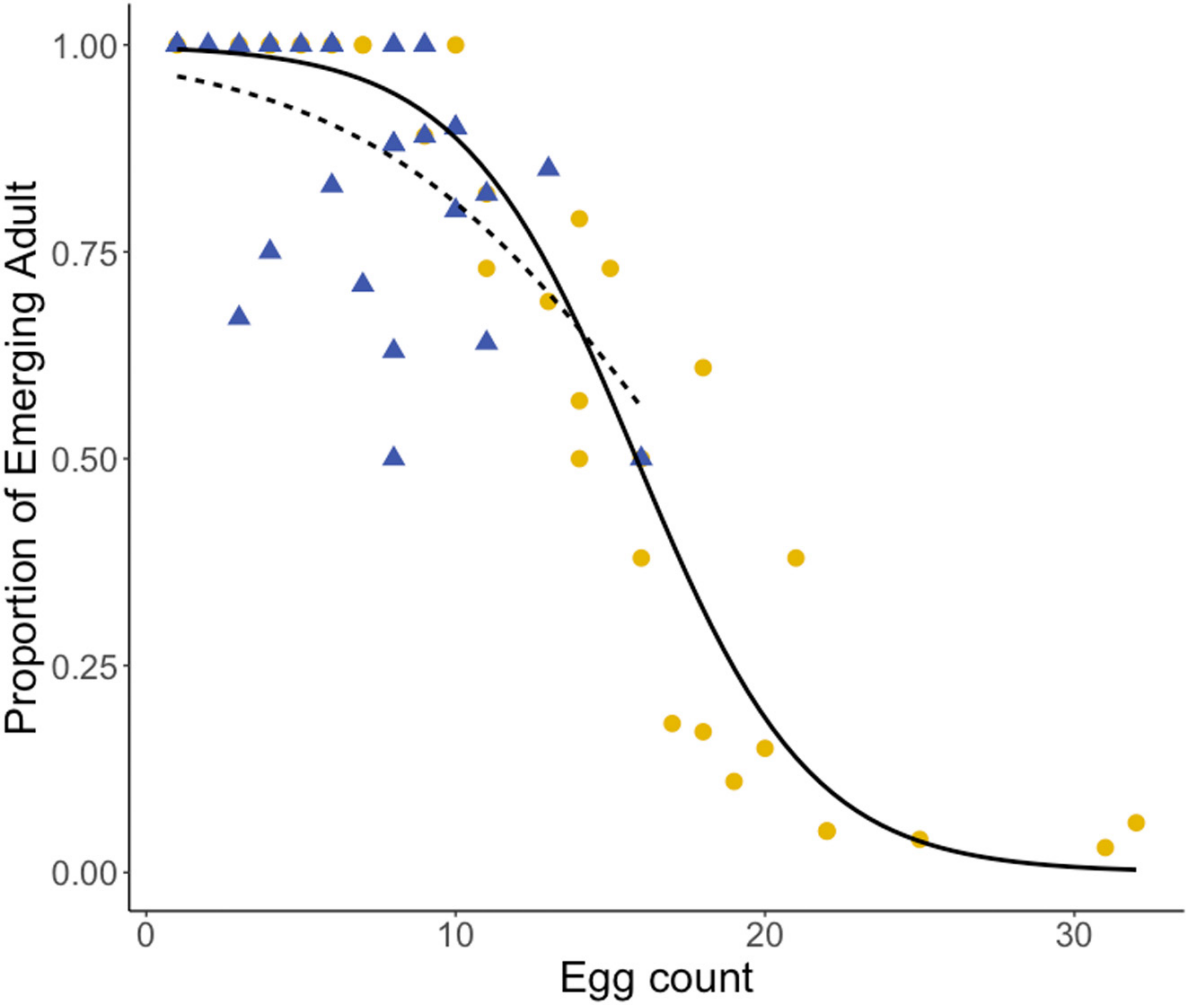
Egg‐to‐adult survival as a function of the number of eggs laid by single *Drosophila melanogaster* females that were exposed previously to different periods of starvation. The yellow points and blue triangles represent egg‐to‐adult survival in the treatments where ovipositing females were starved for 6 and 24 h, respectively. The solid and the dashed lines represent the results of the logistic generalized linear regression model for the 6 and 24 h starvation treatment, respectively.

The eggs from a second, similarly treated cohort of *D. melanogaster* females were allowed to hatch and the larvae to develop to the adult stage. The proportion of surviving offspring to adulthood depended significantly on the number of eggs that were oviposited into the same patch (Figure 9). There was a negative effect of egg count on egg‐to‐adult survival (*F*_1,58_ = 140.9, *p* < .001) and this could be due to increased competition for food and space in crowded cultures as population density increases (Bierbaum et al., 1989). In the additive model, the starvation treatment had no significant effect on survival (*F*_1,57_ = 1.182, *p* = .282).

### 3.5 Feces as a source of heritable symbiont fungi

**FIGURE 10:**
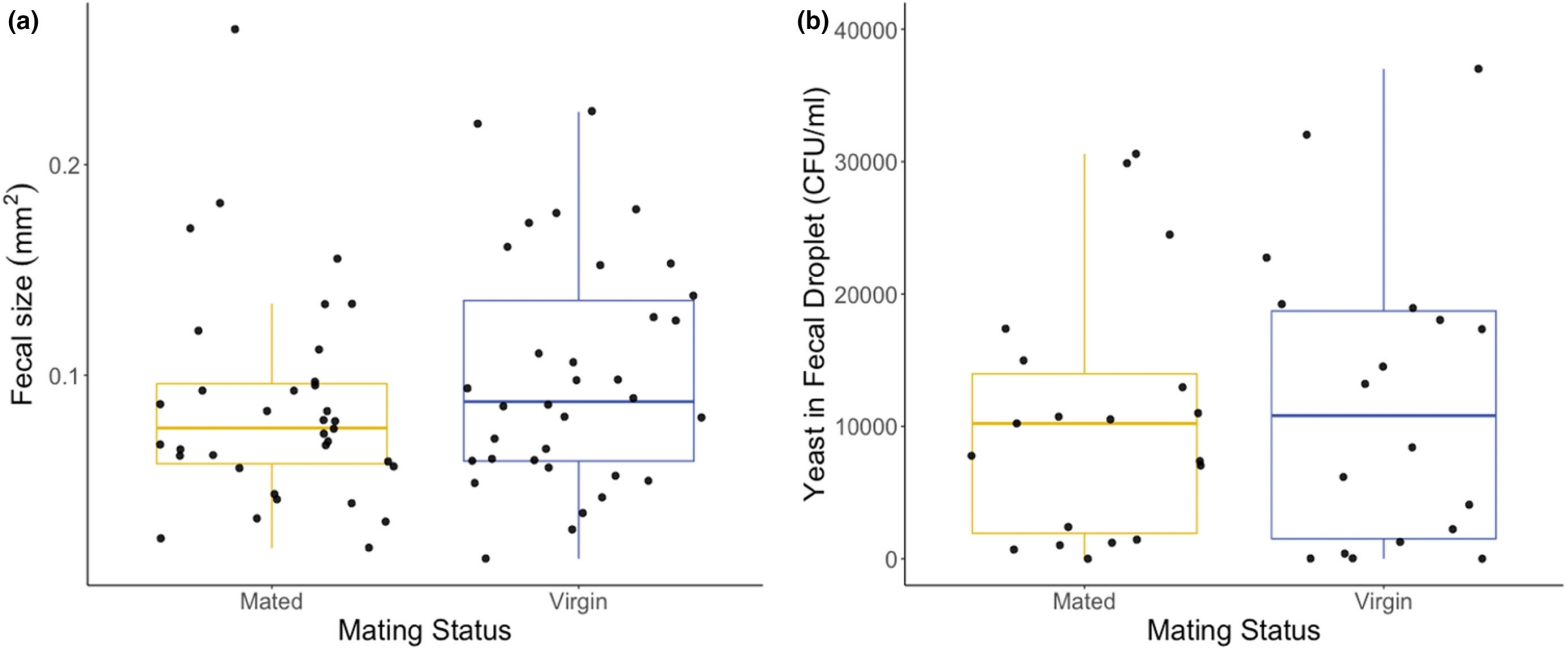
(a) Box plot with jitter showing the relationship between the fecal size and the mating status of the female flies. (b) Box plot with jitter showing the amount of yeast found in fecal droplets by mated and virgin female flies.

In the last experiment, we asked whether the fecal deposition during a female's patch visit contains viable *H. uvarum* cells providing a route of transfer of maternal microbiota to the next generation in *D. melanogaster*. Random picking of single fecal droplets from mated and virgin females (see Experiment 4) revealed strong variation in the amount of yeast transferred via these deposits (Figure 10). The data structure proved unsuitable for adequate statistical modeling, probably because the 69 samples are not truly random in terms of possible inter‐individual variation in the total amount of fecal droplets and the distribution of yeast cells across the deposits of individual females. Using an unpaired Wilcoxon rank sum test, we did not find a difference in the size of fecal material between mated and virgin females (*p* = .262), nor did the number of estimated yeast cells differ between deposits from mated and virgin ones (*p* = .852). Nevertheless, these data clearly show that the feces of fruit flies can contain copious amounts of viable yeast cells, which can then colonize the developmental habitat of the fly larvae.

## 4 DISCUSSION

Simple forms of insect‐fungus mutualism and primal parental care must have preceded the evolution of advanced mutualism‐sociality feedbacks (Biedermann & Rohlfs, 2017). Building on the argument that habitat stability is a major ecological factor determining the fitness payoff of maternal investment in the transfer of symbionts, we proposed a null hypothesis (Figure 1) for insects using ephemeral breeding sites, which rely on fungal ecto‐symbionts but display no obvious parental care or social life. Breeding site ephemerality is assumed to prevent mutualism‐sociality feedback to run its course, which would otherwise promote increasingly specialized symbioses, that is, fungal farming, and complex social life. Upon rejection of the null hypothesis, alternative hypotheses (Figure 1) would open a new perspective on the initial stages of the mutualism‐sociality feedback by adopting the parental care framework.

Here, we found that offspring production, that is, egg‐laying, of *D. melanogaster* females positively correlates with the amount of yeasts transferred to egg‐laying sites (Figure 5). The dietary yeast community in the fly population we kept on raspberry, consisted of a small number of species that have previously been found to be associated with fruit‐inhabiting *Drosophila* (Scheidler et al., 2015), which in our experiment (see Experiment 2) proved to be beneficial to larvae development regardless of their species identity (Figure 6). This result is consistent with the previous studies that *D. melanogaster* are generalists and that they have not developed a specific association with yeast fungi (Anagnostou et al., 2010; Koerte et al., 2020).

Yeast transfer by *Drosophila* flies has also been observed previously (Buser et al., 2014; Quan & Eisen, 2018) but has not been linked to variation in the patch use of individual flies. The extent to which yeasts were transferred in our experiment strongly correlated with patch residence time and a proxy for fungal symbiont load (Figure 5), which supports scenario 2 outlined in Figure 1. In Experiment 1, the amount of yeast transferred to the patch did not correlate with the total number of eggs laid but rather with the presence or absence of eggs on the patch (Appendix I, Table A1) and this may be due to the lack of strong reciprocal specialization in the fly‐yeast association. Egg‐laying site selection in *Drosophila* follows a predictable sequence of behavioral motor programs (Yang et al., 2008); however, a putative “yeast transmission” program may either be only weakly expressed, or its efficiency may be compromised by conflicts between allocating yeast to offspring provisioning or consumption by adults. Interestingly, when *D. melanogaster* females are mono‐associated with *H. uvarum*, we do see a positive relationship between the number of eggs laid and the amount of yeast transmitted. This suggests that the flies at least have the potential to regulate the transmission of microbes in a more sophisticated way than we observed in the first experiment. It is possible that the composition of the microbiota of individual flies determines the amount of viable microbes deposited during oviposition, due to mechanisms yet to be elucidated.

Upon ingestion, food material always enters the crop and is subsequently released into the midgut (Hadjieconomou et al., 2020). Dependent on the hunger status and the capacity to expand and to hold the food material, the crop may serve as a storage organ for microbial symbionts (Pais et al., 2018; Stoffolano & Haselton, 2013). As predicted, we found the crop to harbor viable *H. uvarum* cells. The amount of yeast cells strongly correlated with the size of the crop as determined by the mating status. Crop size negatively correlated with the number of mature oocytes in the ovaries, with mated females having a lower egg load. Due to neurophysiological feedback mechanisms, mated females tend to have more expanded crops containing more food material than virgin ones (Hadjieconomou et al., 2020). While this is in line with our hypothesis that mated females store more yeasts in their crops, we cannot exclude the possibility that the observed reciprocal size changes in ovaries and the crops are due to physical constraints of the female abdomen. When the ovaries are full of mature eggs in the virgin females, the crop may be limited in its capacity to expand and thus the ability to ingest yeasts. Only when the ovaries carry fewer mature oocytes or are empty, like in the mated females that laid eggs, can the crop be expanded further into the abdomen. Regardless of the mechanisms underlying the variation in crop size and the amount of yeast that they can potentially carry, egg‐laying females may harbor more yeast than non‐egg‐laying females. This corresponds well with the finding that reproduction is accompanied by more intensive deposition of viable yeast cells that support the development of *Drosophila* larvae.

Indeed, we observed successful although density‐dependent egg‐to‐adult survival from clutches laid by females, mono‐associated with *H. uvarum*, which demonstrates the link between the crop as a storage organ for yeast and the provisioning of the larval offspring with dietary symbionts. Even though enforced fasting reduces the expansion of the crop and the amount of viable *H. uvarum* cells transmitted to the egg‐laying site, it did not severely impair larval survival to the adult stage. Therefore, despite a temporary lack of opportunities to replenish their symbiont storage, females are still able to provide their offspring with a sufficient amount of yeast. This is consistent with previous observations that exceptionally low levels of initial yeast cell masses can support *D. melanogaster* development well (Anagnostou et al., 2010).

In many insect taxa, adult regurgitation and defecation are major paths of transgenerational transfer of microbes beneficial to offspring fitness (e.g., Fukatsu & Hosokawa, 2002; Körner et al., 2016; Parker et al., 2019; Rosengaus et al., 2013). We provide direct evidence that single units of the fecal material deposited by *D. melanogaster* can contain a high amount of viable *H. uvarum* cells, which supports larval development as mentioned before. How covariation in symbiont transfer and insect reproduction is regulated, for example, whether the individual fecal droplets of egg‐laying females contain higher amounts of yeast, or the total amount of excrement is higher, remains to be investigated.

In our experiments, we cannot always distinguish between cause and effect; for example, the observed relationship between yeast transmission and reproduction is correlative. Reciprocally beneficial traits in interactions between insects and fungi need not necessarily have coevolved but could have evolved independently; and the evolutionary innovations associated with the proposed feedback loop between mutualism and sociality could have arisen as exaptation, that is, as by‐products of the adaptive evolution in other selective traits (Gould & Vrba, 1982). Several of such non‐coevolved traits seem to make many yeasts and insects ideal partners (Blackwell, 2017; Stefanini, 2018). The fact that different yeast fungi attract *Drosophila* flies via a similarly broad spectrum of volatile metabolites and have comparable effects on larval development (Anagnostou et al., 2010; Palanca et al., 2013; Quan & Eisen, 2018; Scheidler et al., 2015; this study) suggests that *Drosophila*‐yeast associations are characterized by low levels of partner choice and fidelity. Low partner fidelity likely indicates the lack of specialized mutualisms including dedicated symbiont transmission by *Drosophila* females (see Kaltenpoth et al., 2014 for consequences of higher partner fidelity).

A plethora of taxa within the family Diptera settle on ephemeral breeding substrates and rely on microbial symbionts, but no Dipteran group appears to have evolved eusociality or any known advanced form of social life, such as termites within the Blattodea (Inward et al., 2007). However, numerous social interactions and collective behaviors within one generation have been described recently, particularly in *Drosophila* (Chen & Sokolowski, 2022; Dombrovski et al., 2017; Durisko et al., 2014; Lihoreau et al., 2016). Moreover, *Drosophila* flies are known to be sub‐social, that is, they aggregate (e.g., McKinney et al., 2021; Philippe et al., 2016). Aggregation is mediated by the emission of pheromonal compounds from fecal deposits (Keesey et al., 2016), which may additionally be enhanced by the attraction of flies to yeast volatiles (Günther & Goddard, 2019). Therefore, the enhanced transfer of yeast symbionts would not only benefit their own offspring but also the offspring of females joining fly aggregations or are attracted to the egg‐laying sites of other females, and vice versa. Aggregation of egg‐laying females may thus be an adaptive response that increases per capita offspring fitness due to yeast‐mediated breeding site amelioration (Rohlfs & Hoffmeister, 2003). Positive insect–fungus interactions may thus improve conditions for symbiosis stability and consequently strengthen conditions for the sociality‐mutualism feedback to begin and to run its course (Biedermann & Rohlfs, 2017).

## AUTHOR CONTRIBUTIONS

**Hanna Cho:** Conceptualization (equal); data curation (lead); formal analysis (lead); investigation (lead); methodology (lead); validation (lead); visualization (lead); writing – original draft (lead); writing – review and editing (equal). **Marko Rohlfs:** Conceptualization (equal); formal analysis (supporting); methodology (supporting); writing – original draft (supporting); writing – review and editing (equal).

## FUNDING INFORMATION

University of Bremen funds the research and the publication charge (Golden Open Access).

## Data Availability

The data that support the findings of this study are openly available in Dryad at https://doi.org/10.5061/dryad.ns1rn8pzp.

## I FULL MODELS AND MAMS OF STATISTICAL ANALYSIS

### Yeast transmission by individual *Drosophila melanogaster* females

**TABLE A1:** Results of an ANOVA for the GLM (Gaussian, identity link) containing the factors affecting the amount of yeast that individual *Drosophila melanogaster* females shed during a single visit to a raspberry agar medium, and all the interaction terms are shown.

Factor	Sum sq	df	*F* value	Pr (>*F*)
log (Egg + 0.1)	0.020	1	0.0115	.915
log (Yeast load + 0.1)	60.039	1	34.80	<.001
log (Time + 0.1)	24.397	1	14.14	<.001
Body size	2.575	1	1.492	.225
Presence or absence of eggs	4.726	1	2.739	.102
log (Egg + 0.1):log (Time + 0.1)	0.255	1	0.1480	.701
log (Egg + 0.1):Body size	0.917	1	0.5316	.468
log (Yeast load + 0.1):Body size	0.429	1	0.2485	.619
Residuals	141.451	82		

**TABLE A2:** Results of an ANOVA for the GLM (Gaussian, identity link) containing the factors affecting the amount of yeast that individual *Drosophila melanogaster* females shed during a single visit to a raspberry agar medium. Only the results of the reduced minimal adequate model are shown.

Factor	Sum sq	df	*F* value	Pr (>*F*)
log (Yeast load + 0.1)	64.723	1	38.54	<.001
log (Time + 0.1)	41.374	1	24.64	<.001
Presence or absence of eggs	35.662	1	21.23	<.001
Residuals	146.089	87		

### Transmitted yeast fungi as dietary symbionts

**TABLE A3:** Results of an ANOVA for the GLM (binomial, logit‐link) containing the factor affecting the survival of *Drosophila melanogaster* larvae to adulthood. The Full model is the MAM in this case.

Factor	df	*χ* ^2^ value	Pr (>*F*)
Treatment	6	20.905	<.01

### The crop as a potential storage organ of symbiotic yeast fungi

**TABLE A4:** Results of an ANOVA for the GLM (Gamma, log‐link) containing the factors affecting the size of a crop in *Drosophila melanogaster* females.

Factor	Sum sq	df	*F* value	Pr (>*F*)
Oocyte	0.49301	1	2.927	.105
Body size	0.00947	1	0.0562	.815
Yeast in crop	1.74780	1	10.38	<.01
Mating status	0.31826	1	1.889	.187
Oocyte: Body size	0.27230	1	1.617	.221
Oocyte: Yeast in crop	0.00003	1	0.0002	.989
Oocyte: Mating status	0.10196	1	0.6054	.447
Body size: Yeast in crop	0.10779	1	0.6400	.435
Body size: Mating status	0.09103	1	0.5404	.472
Yeast in crop: Mating status	0.12936	1	0.7680	.393
Residuals	2.86329	17		

*Note*: All the results from the main and interaction terms are shown.

**TABLE A5:** Results of an ANOVA for the GLM (Gamma, log‐link) containing the factors affecting the size of a crop in *Drosophila melanogaster* females. Only the results of the reduced minimal adequate model are shown.

Factor	Sum sq	df	*F* value	Pr (>*F*)
Yeast in crop	2.9618	1	12.44	<.01
Mating status	2.1069	1	8.851	<.01
Residuals	5.9508	25		

### Effect of fasting on yeast transmission and development success of the offspring

#### Part 1

**TABLE A6:** Results of an ANOVA for the GLM (quasi‐Poisson, log‐link) containing the factors affecting the amount of yeast *Drosophila melanogaster* females in different starvation periods transmit during egg‐laying.

Factor	Sum sq	df	*F* value	Pr (>*F*)
Egg	3151.1	1	27.07	<.001
Starvation period	3066.8	1	26.34	<.001
Residuals	6636.4	57		

*Note*: The Full model is the MAM in this case.

#### Part 2

**TABLE A7:** Results of an ANOVA for the GLM (quasi‐binomial, logit‐link) containing the factors affecting the survival of *Drosophila melanogaster* from egg to adult.

Factor	Sum sq	df	*F* value	Pr (>*F*)
Egg	19.7676	1	126.5	<.001
Starvation period	0.2136	1	1.366	.247
Egg: Starvation period	0.5801	1	3.711	.059
Residuals	8.7537	56		

*Note*: The eggs were laid by females that were treated with different starvation periods. All the results from the main and interaction terms are shown.

**TABLE A8:** Results of an ANOVA for the GLM (quasi‐binomial, logit‐link) containing the factors affecting the survival of *Drosophila melanogaster* from egg to adult.

Factor	Sum sq	df	*F* value	Pr (>*F*)
Egg	26.188	1	140.9	<.001
Residuals	10.784	58		

*Note*: The eggs were laid by females that were treated with different starvation periods. Only the results of the reduced minimal adequate model are shown.

## II IDENTIFICATION OF YEASTS TRANSMITTED BY *DROSOPHILA MELANOGASTER* FEMALES

To assess the identity of yeasts transmitted and to have isolates available to quantify their role as dietary mutualists for *D. melanogaster* development on raspberries, we selected 33 colonies out of the 91 replicates based on their differences in size and colony morphology. The isolates were again sub‐cultured in a liquid medium for 48 h at 25°C. The liquid cultures were then used for DNA‐based identification.

Yeasts were identified by amplifying and sequencing the ITS region using the primer pair ITS1F (Gardes & Bruns, 1993) and ITS4 (White et al., 1990). Polymerase chain reactions (PCRs) were performed with a 20 μL volume, which contained the following: 2 μL of template DNA (50 ng/μL), 2 μL 10× PCR buffer, 0.4 μL of a 10 μM stock solution of each primer (ITS1F primer and ITS4 primer), 0.4 μL of 10 mM dNTP's mix, and 0.1 μL of Taq DNA polymerase. The PCRs were run with a temperature profile of 4 min at 94°C, followed by 30 cycles of 30 s at 94°C, 30 s at 50°C, and 90 s at 72°C. The 30 cycles were followed by 10 min at 72°C. Gel electrophoresis was used to confirm the presence of DNA in each sample and gel documentation was viewed to confirm that the length of the DNA fragments was appropriate for the amplified ITS region. A 5 μL aliquot of each PCR product and a negative control were run on a 1.5% agarose gel and were photographed under a UV light. Only 27 samples with a clear presence of DNA from this documentation and 1 positive control (*S. cerevisiae*) were sequenced.

The PCR products were purified with a FastGene GEL/PCR extraction kit and the quality and concentration of the sample DNA were measured with a spectrophotometer. The purified PCR products were then Sanger‐sequenced at GATC biotech. The sequences were analyzed with the BLAST standard nucleotide‐nucleotide basic local alignment search tool (http://www.ncbi.nlm.nih.gov/BLAST/). The BLAST parameters were set as default and the outputs from the BLAST searches were sorted based on the maximum identity and were recorded. Synonyms or teleomorphs of other genus or species names were cross‐referenced using NCBI taxonomy and MycoBank database (http://mycobank.org). Sequence‐based identities with a cutoff of 93% or greater and a query cover >88% were considered reliable. The best hit was selected by the sequence with the highest maximum identity to the query sequence.

**TABLE A9:** Identification of the yeast isolates with the best blast hit against NCBI nucleotide collection database.

Sample ID	Best BLAST hits	Accession	Fragment length [bp]	Query cover [%]	Identity [%]	*E*.value	Score
SC	*Saccharomyces cerevisiae*	HG532088.1	322	100	97	1e‐154	556
F1	*Wickerhamomyces pijperi*	KY105912.1	100	77	96	6e‐27	130
F2	*Wickerhamomyces pijperi*	KY105912.1	121	98	93	1e‐39	172
F6	*Wickerhamomyces pijperi*	KY105912.1	126	96	95	4e‐45	191
F13‐1	*Pichia terricola*	KY104650.1	412	99	99	0	732
F13‐2	*Wickerhamomyces pijperi*	KY105912.1	100	97	95	4e‐34	154
F15	*Wickerhamomyces pijperi*	KY105912.1	111	99	94	2e‐38	169
F17	*Wickerhamomyces pijperi*	KY105912.1	97	97	94	6e‐32	147
F26	*Wickerhamomyces pijperi*	KY105912.1	116	95	93	6e‐38	167
F31	*Wickerhamomyces pijperi*	KY105912.1	113	96	93	7e‐37	163
F33	*Wickerhamomyces pijperi*	KY105912.1	115	96	95	1e‐39	172
F65	*Hanseniaspora uvarum*	KY103557.1	176	96	99	4e‐81	311
F89	*Wickerhamomyces pijperi*	KY105912.1	105	94	96	2e‐36	161
F97	*Wickerhamomyces pijperi*	KY105912.1	126	97	95	3e‐46	195
F99	*Wickerhamomyces pijperi*	KY105912.1	113	97	94	6e‐38	167
P4	*Wickerhamomyces pijperi*	KY105912.1	89	84	99	1e‐28	135
P10	*Wickerhamomyces pijperi*	KY105912.1	143	96	91	1e‐46	196
P14	*Wickerhamomyces pijperi*	KY105912.1	472	99	95	0	754
P28	*Pichia terricola*	KY104650.1	400	99	99	0	725
P34	*Wickerhamomyces pijperi*	KY105912.1	578	99	94	0	887
P38	*Hanseniaspora uvarum*	KY076617.1	727	99	99	0	1310
P42	*Pichia terricola*	KY104650.1	399	98	100	0	730
P50	*Hanseniaspora uvarum*	KF728793.1	670	100	99	0	1208
P51	*Wickerhamomyces pijperi*	KY105912.1	474	98	96	0	782
P62	*Hanseniaspora uvarum*	KY103557.1	179	97	98	4e‐81	311
P68	*Hanseniaspora uvarum*	KY366247.1	174	100	98	2e‐80	309
P77	*Hanseniaspora uvarum*	GU237050.1	218	88	98	7e‐90	340
P92	*Hanseniaspora uvarum*	KY103557.1	189	96	98	8e‐84	320
